# Surgical management of medial and lateral elbow instability secondary to acute atypical complex elbow dislocation: Case report and literature review

**DOI:** 10.1016/j.ijscr.2021.01.024

**Published:** 2021-01-15

**Authors:** Talal Almalki, Abdullah Y. AlMarshad, Khalid Beidas, Khaled Alshurafa, Hamad Al Bassam

**Affiliations:** a Security Forces Hospital Program, Department of Orthopedics, Riyadh, Saudi Arabia; b King Faisal Specialist Hospital and Research Centre, Department of Orthopedics, Riyadh, Saudi Arabia; c Prince Muhammad Bin Abdul-Aziz, Department of Orthopedics, Riyadh, Saudi Arabia; d Ministry of Health, Department of Emergency Medicine, Saudi Arabia

**Keywords:** LCL, lateral collateral ligament, MCL, medial collateral ligament, MRI, magnetic resonance imaging, ROM, range of motion, Elbow dislocation, Lateral elbow instability, Medial elbow instability

## Abstract

**Introduction and importance:**

Elbow dislocation is common in adults, and complex elbow dislocations are generally associated with bone fractures. Anteromedial coronoid fracture, in association with lateral collateral ligament (LCL) disruption, often results from varus posteromedial forces. “Terrible triad” injuries are more likely to result from valgus posterolateral forces. However, our case presentation has combined medial and lateral elbow instability in addition to “terrible triad” injury of the elbow with no radial head injury.

**Case presentation:**

The patient was a 38-year-old man with an atypical complex elbow dislocation. He was successfully treated by stabilizing the medial epicondyle and coronoid anterolateral facet fractures, in addition to LCL repair and medial collateral ligament (MCL) reconstruction. A radial head fracture was unnoted. The procedure yielded satisfactory functional outcome, with a stable and painless full elbow range of motion.

**Clinical discussion:**

Multi-ligament injuries with coronoid fractures result in highly unstable elbow joints, forming a variant of the “terrible triad” injury. Surgical options vary according to the surgeon’s experience and equipment availability. In this case, direct LCL repair and MCL reconstruction were performed and were well tolerated. Elbow stability improved and the patient experienced improved functionality with minimal pain. However, it may be premature to report a definite outcome in this case because of short follow-up time postoperatively.

**Conclusion:**

The injury described in this case has a unique presentation as a multi-ligamentous injury will make the elbow very unstable. Thus, careful clinical judgment, knowledge, and experience are needed to identify the underlying injury and for optimal management.

## Highlights

• The patient presented with an atypical complex elbow dislocation with fracture.
• The patient underwent lateral collateral ligament repair.
• The patient also underwent medial collateral ligament reconstruction.
• Elbow stability improved and the patient experienced improved functionality.
• Care is needed to identify and manage the underlying injury in this type of case.

## 1 Introduction

Elbow dislocation is common in adults and about 20% of cases are fracture-related; these are known as complex elbow dislocations [1]. Differences in the direction of force to the elbow result in varying fracture patterns [2]. Particularly, the valgus postero-lateral forces tend to cause “terrible triad” injuries [2]. Although varus posteromedial forces are believed to cause anteromedial coronoid fractures in association with lateral collateral ligament (LCL) disruption, they are not considered to be associated with medial collateral ligament (MCL) or radial head injuries [2]. Elbow injury progression moves from lateral to medial, as the LCL tends to fail first, while the MCL fails last [3]. It is thus essential to ascertain the exact bony and soft tissue damage involved in any elbow injury both clinically and radiologically [2].

To achieve elbow stability, it is important to treat LCL disruption and any radial head fracture [4]. Additionally, coronoid fracture fixation is typically performed for type II and III injuries [4], where treatment aims to stabilize the elbow and restore a functional range of motion (ROM) [2]. Our report describes a case of acute elbow dislocation that developed into an atypical complex elbow injury resulting in medial and lateral elbow instability. The patient, managed in a subspecialized centre, underwent surgical bony and ligamentous stabilization, which resulted in a good functional outcome. This suggests that when addressing medial instability by reconstructing the ulnar collateral ligament with autografting, ulnar nerve transposition and stabilization are effective in restoring elbow medial stability, allowing a faster return to pre-injury levels [5].

This case report was prepared in line with the SCARE criteria [6].

## 2 Presentation of case

The patient was a right-handed 38-year-old Saudi male soldier. He was a smoker but had no history of chronic medical diseases or surgery not on any chronic medications and no significant family history. He arrived at the emergency department with a cast on the left upper extremity, applied outside of the institution. Injury occurred after a fall on his outstretched hand. In the local hospital, he was diagnosed with isolated acute elbow dislocation, which was managed with closed reduction and splinting. Distal neurovascular examination appeared intact pre- and post-reduction, and there were no open wounds. The patient was referred for follow-up in a subspecialized center, and thus came 1 day post-injury for consultation at the trauma orthopedic outpatient clinic.

The patient reported having an injury 15 years ago on the same side. He was splinted for 14 days; however, he could not remember the exact diagnosis. On examination, he was unable to bear weight on the left upper limb. The splint was removed for examination and reapplied afterwards. During the inspection, elbow swelling and diffuse tenderness were noted. Painful ROM from 30°–130° (elbow stable, active-assisted), but was full passively. Varus and valgus tests reproducing pain laxity were unnoted because of the examinations’ intrinsic limitations, and neurovascular examination intact.

**Fig. 1:**
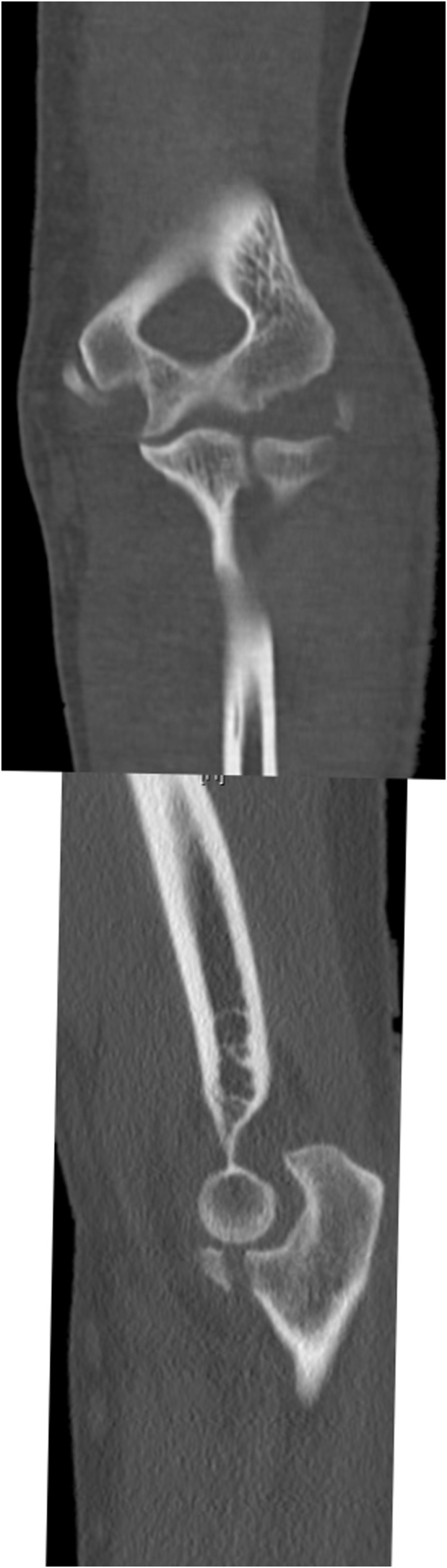
Coronal and sagittal CT scan of the left elbow showed avulsion fracture from the medial and lateral epicondyles of the distal humerus in addition to type 2 coronoid fracture.

Left-elbow anteroposterior and lateral radiographs revealed a coronoid fracture with a Regan and Morrey classification grade 2, including a capitellum fragment, suggested an old avulsion. Additionally, a medial fragment appeared as an avulsion from the trochlea, and elbow joint subluxation was noted. Computed tomography (coronal and sagittal views; Fig. 1) showed a type 2 coronoid fracture, with an avulsion fracture from the medial and lateral epicondyles of the distal humerus.

**Fig. 2:**
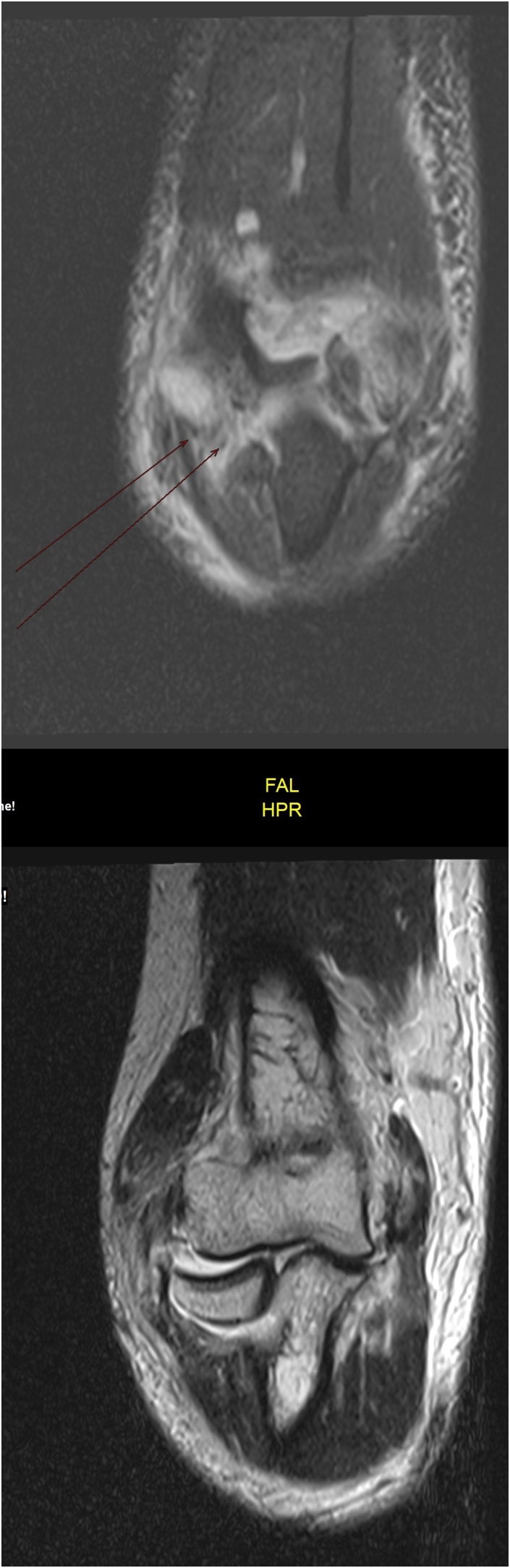
Left elbow T1, T2 weighted MRI showed a coronoid fracture, medial epicondylar fracture, and radial and ulnar collateral ligaments tear.

After clinical and radiological examination, ligamentous injury was suspected. Considering the COVID-19 pandemic, a decision (with patient consent) was made to admit the patient for magnetic resonance imaging (MRI) and surgery, if necessary. After 10 days, MRI was performed T1, T2 MRI (Fig. 2) showed a coronoid fracture, medial epicondylar fracture, and radial and ulnar collateral ligament tear.

After reviewing the case, surgery performed by a senior consultant orthopedic surgeon who subspecialized in trauma more than 20 years, included direct LCL repair, open reduction of the anterolateral coronoid fracture, internal fixation, MCL reconstruction with a hamstring autograft, and ulnar nerve decompression using anterior transposition and evaluation of elbow joint stability under general anesthesia.

After general anesthesia was applied, the lateral pivot shift test (O’Driscoll method [7]), was performed with the patient’s arm overhead; this was positive. Moreover, a positive lateral stress test was noted [8]. The surgery was then performed with the patient supine and supported by an arm table. The elbow was approached using two separate lateral and medial incisions. After subcutaneous anterior transposition of the ulnar nerve, the lateral structure was exposed through the Kocher interval and an avulsed LCL complex, including the common extensor from the lateral epicondyle, was noted; the radial head was intact. The LCL complex was then repaired with a non-absorbable Ethibond suture anchor Number 2, locked at the origin of the tendon and ligament [8].

After lateral stabilization, a valgus stress test was performed, and instability was noted. The contralateral hamstring autograft, obtained by another surgical team, another incision was made approaching the medial side of the elbow, and an MCL mid-rupture was found with a notable gap (>2 cm). Reconstruction with the hamstring autograft commenced; stability testing performed intraoperatively showed stable varus and valgus instability. Patient tolerated the procedure with no immediate post-operative complications.

**Fig. 3:**
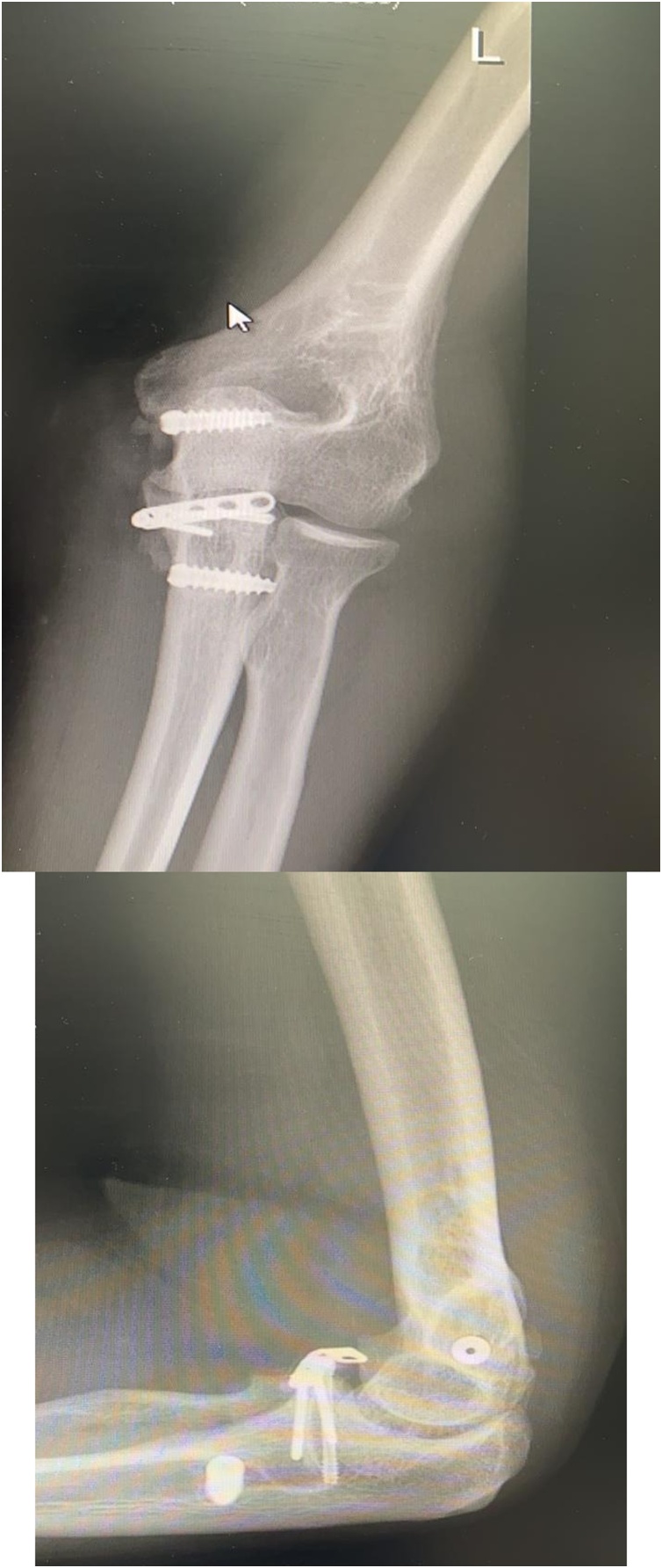
3-month post operative AP and lateral elbow x ray showed Plating of the coronoid fracture and anchor screws fixing the MCL autograft.

**Fig. 4:**
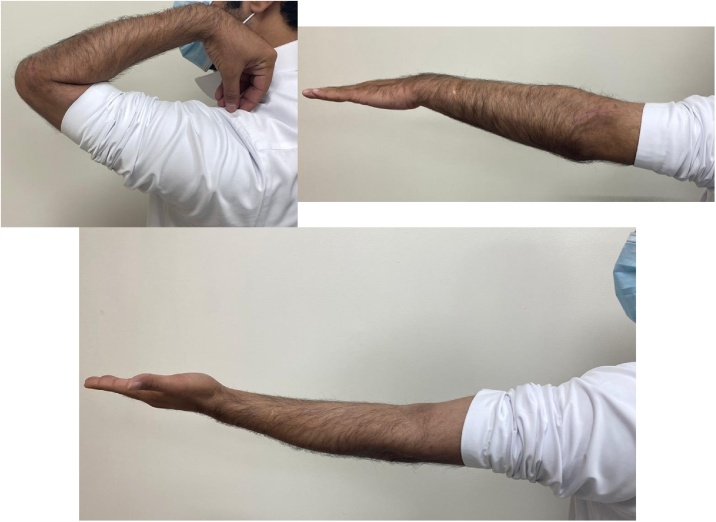
3-month follow-up visit at the clinic, with painless left elbow and full ROM.

Postoperatively the elbow was protected by an above-elbow splint with the forearm held in a neutral position for 2 weeks. A hinge brace applied for the next 4 weeks to allow a gradual weekly 10° decrease in flexion arc. The patient then started passive and assisted-active ROM exercises. In a 3-month follow-up visit at the clinic, an elbow x ray obtained (Fig. 3), the patient presented with a painless elbow and full ROM (Fig. 4), at this point patient can share his perspective after received the treatment.

## 3 Discussion

Despite the frequency of elbow dislocations, Josefsson and Nilson discovered that the medial epicondylar fracture was the most common fracture associated with elbow dislocation; this was confirmed in this case. In order of frequency, other associated injuries include the radial head, lateral epicondyle, coronoid process, capitellum, and olecranon process [9]. According to Jones and Jordan, the management of acute elbow complex dislocations should begin with immediate closed reduction, followed by a complete clinical examination of the elbow, including computed tomography [1].

In a review article on the management of elbow “terrible triad” injuries, Mathew suggested that MCL repair was unnecessary where the elbow became stable after repair of the coronoid process, radial head, and LCL from 30° to a full flexion ROM [10]. Geli et al. similarly conducted a study analyzing varus posteromedial rotatory instability and emphasized the importance of repairing the LCL and the anteromedial facet of the coronoid, where the MCL is initially attached [4].

In a prospective randomized study by Josefsson et al., which analyzed 30 simple elbow dislocations without associated fractures, all patients experienced both LCL and MCL rupture. However, no significant differences were observed between surgical and non-surgical treatments [11].

Our report describes a complex acute elbow dislocation associated with both MCL and LCL rupture along with medial epicondylar and coronoid anterolateral facet fractures, but without radial head fracture. Although the patient had both osseous and ligamentous injuries, this case could not be classified as a “terrible triad” injury of the elbow because of the absence of radial head injury. Thus, this case is a rare presentation with this kind of complexity.

Shukla et al. described a new surgical technique for bilateral congenital elbow atraumatic instability in pediatric cases. They reported a case of varus and posterolateral elbow instability, discovered intraoperatively, which was treated with staged LCL reconstruction and internal fixation, resulting in a stable elbow [12].

Ramzi et al. also reported a case where elbow dislocation had an unusual combined presentation that was classified as ranging between a terrible triad injury and an Essex-Lopresti injury. The case was treated with closed reduction of the elbow and distal radioulnar joint, followed by MCL and LCL repair, resulting in a stable elbow without coronoid fracture fixation [13].

Patiño et al. reported two cases of childhood post-traumatic cubitus varus complicated with posterolateral rotatory instability which were treated with distal humerus valgus osteotomy and ligament reconstruction. This resulted in full ROM restoration, stable elbow, and satisfactory functional outcomes [14].

Zlowodzki et al. conducted a meta-analysis of randomized controlled trials to compare the results of simple ulnar nerve decompression and anterior ulnar nerve transposition. They found no difference in motor nerve conduction velocity or clinical outcomes between the two groups [15]. In the current case, anterior ulnar nerve transposition was performed to prevent ulnar nerve irritation. The limitations of our case study include the short term follow up, which might limit the generalizability of the findings to longer period. Further cases with longer follow-up could be done to overcome these limitations.

## 4 Conclusion

The mechanism of elbow injury is the main determinant of injury pattern and fracture configuration. Injuries, as seen in this case, are uncommon and are challenging to treat. A multi-ligament injury with coronoid fracture results in a highly unstable elbow. Surgical options vary according to the surgeon’s experience and equipment availability. In the current case, it seems premature to comment on the final outcome as the follow-up period has been minimal. However, clinically, the elbow was stable and the patient presented with little functional disability and minimal pain. As this type of injury offers a presentation similar to that of a “terrible triad” injury, care is needed to identify the underlying injury and for optimal management.

## Note

There are no patient details (name or medical record number), or institution name included in the figures.

## Declaration of Competing Interest

None.

## Funding

None.

## Ethical approval

The study was approved by institutional review board in accordance with the national committee of bio ethics guidelines.

## Consent

Written informed consent was obtained from the patient for publication of this case report and accompanying images. A copy of the written consent is available for review by the editor-in-chief of this journal on request.

## Author contribution

Talal Almalki contributes the paper with Supervision, data analysis and interpretation.

Abdullah Y. AlMarshad contributes the paper with writing the paper and data analysis.

Khalid Beidas contributes the paper with data collection, data analysis and interpretation.

Khaled Alshurafa contributes the paper with data analysis, interpretation and whole management.

Hamad Al Bassam contributes the paper with data collection, data analysis and interpretation.

## Registration of research studies

Not Applicable.

## Guarantor

Abdullah AlMarshad.

## Provenance and peer review

Not commissioned, externally peer-reviewed.
